# Proximate composition, lipid and elemental profiling of eight varieties of avocado (*Persea americana*)

**DOI:** 10.1038/s41598-023-50119-y

**Published:** 2023-12-20

**Authors:** Chaimae Nasri, Yasmina Halabi, Ahmed Hajib, Hasnae Choukri, Hicham Harhar, Learn-Han Lee, Vasudevan Mani, Long Chiau Ming, Khang Wen Goh, Abdelhakim Bouyahya, Mohamed Tabyaoui

**Affiliations:** 1 Laboratory of Materials, Nanotechnology, and Environment LMNE, Faculty of Sciences, Mohammed V University of Rabat, BP 1014, Rabat, Morocco; 2 Higher School of Education and Training (ESEF), Université Ibn Zohr, Agadir, Morocco; 3 International Center for Agricultural Research in the Dry Areas, Rabat, Morocco; 4 Sunway Microbiome Centre, School of Medical and Life Sciences, Sunway University, 47500 Sunway City, Selangor Darul Ehsan Malaysia; 5 Research Center for Life Science and Healthcare, China Beacons of Excellence Research and Innovation Institute (CBI), University of Nottingham Ningbo China, Zhejiang, China; 6 Department of Pharmacology and Toxicology, College of Pharmacy, Qassim University, 51452 Buraidah, Saudi Arabia; 7 Faculty of Data Science and Information Technology, INTI International University, 47500 Nilai, Malaysia; 8 Laboratory of Human Pathologies Biology, Department of Biology, Faculty of Sciences, Mohammed V University in Rabat, Rabat, Morocco

**Keywords:** Biotechnology, Plant sciences, Ecology, Environmental sciences

## Abstract

Eight Moroccan avocado varieties were analyzed for their nutritional composition and physicochemical properties. The nutritional contents of the sample were determined through the evaluation of the moisture, oil, ash, protein, and carbohydrate contents, and energy value calculation. Additionally, macroelements (Ca, Mg, and Na) and microelements (Fe, Zn, Cu, and Mn) were determined in the mineral profile. Oils were examined also for their fatty acid, phytosterol, and tocopherol profiles. As a result of the study, the avocado presents significant differences between the eight studied varieties (p < 0.05), with regard to moisture content (57.88 g/100 g to 84.71 g/100 g), oil content (8.41 g/100 g to 57.88 g/100 g), ash (0.57 g/100 g to 1.37 g/100 g), protein content (5.7 g/100 g to 8.61 g/100 g), carbohydrate content (5.63 g/100 g to 14.61 g/100 g), and energy value (99.9 kcal/100 g to 316.8 kcal/100 g). Sodium (5783.01 mg/kg to 12,056.19 mg/kg) was the predominant macro-element in all varieties, followed by calcium (295.95 mg/kg to 531.67 mg/kg), and magnesium (246.29 mg/kg to 339.84 mg/kg). Copper (85.92 mg/kg to 112. 31 mg/kg) was the main microelement in all varieties, followed by iron (8.5 mg/kg to 20.32 mg/kg), and manganese (7.3 mg/kg to 18.45 mg/kg), while zinc (1.72 mg/kg to 5.66 mg/kg) was detected in small amounts. In addition, significant difference was observed in lipid profiles, according to the eight studied varieties (p < 0.05). Avocado oils were mainly composed of monounsaturated fatty acids (76.89 g/100 g to 84.7 g/100 g), with oleic acid (50.38 g/100 g to 71.49 g/100 g) standing out as particularly characteristic, while β-sitosterol (l2365.58 mg/kg to 4559.27 mg/kg), and α-tocopherol (30.08 mg/kg to 182.94 mg/kg) were among its major phytosterols and tocopherols. All avocado varieties represented in this study can be consumed as a fruit as an excellent source of energy, minerals, fatty acids, phytosterols, and tocopherols. The regular consumption of this fruit provides the body with several essential nutrients.

## Introduction

Currently, foods that are full of bioactive products are highly sought after because they have properties beyond their nutritional value. These foods are called nutraceuticals or functional foods^1,2^. On the other hand, their nutritional value is crucial for maintaining the health of consumers. Among the properties sought in functional foods, antioxidants are the molecules that have added value because they contribute, through the consumption of these foods, to preventing a certain number of pathologies linked to stress, as well as to strengthening the immune system^1,3^.

In Morocco, Lauraceae family contains some remarkable plants used as foods and as traditional remedies. *Persea americana* Mill is one Lauraceae species which is importantly consumed in Morocco and worldwide^1,3^.

The fruits of are rich by different chemical compounds in particularly monounsaturated fatty acids like oleic acid^4^, vitamins, and minerals (like vitamins K, B6 and C, copper, folate and potassium, among others), different polyphenols including perseitol, ferulic, quinic, pantothenic, abscisic, and trans-cinnamic acids as well as catechin^5^. Recent investigations showed that avocado bioactive compounds can exhibit different biological functionalities^2^.

Indeed, monounsaturated fatty acids (in particularly oleic acid especially)^4^ which contribute to good cardiovascular function, and inhibition the growth of prostate cancer cells^6^, inducing the death of breast cancer cells and suppress liver damage^7^. Moreover, with its different vitamins and polyphenols compounds, avocado exhibits remarkable antioxidant properties.

Moreover, avocado is being currently among the fruits with the highest energy intake (169 kcal/100 g) and with a low sugar content, the avocado remains an ideal food source for diabetics^8^. Polyphenols containing in avocado which include ferulic, quinic, pantothenic, abscisic, and trans-cinnamic acids^5^, can limit the oxidative stress that therefore exhibit neuroprotective effect against significant neuronal damage found in amyotrophic lateral sclerosis, Huntington's, Parkinson's, and Alzheimer's disease^9^. It was also revealed that the fruits with the highest energy intake (169 kcal/100 g) and with a low sugar content, the avocado remains an ideal food source for diabetics^8^**.**

Originally from Mexico and Central America^10^, the avocado has also been able to establish itself in the Moroccan market. In less than 10 years, Morocco has become a significant player in the international avocado trade and its exports have exceeded 30,000 tons. Thus, it is the 3rd African exporter behind Kenya and South Africa, is also part of the producing and exporting countries that benefit from this craze. The proximity of Europe with only four days of transit, and the high quality of avocados make Morocco an interesting supplier compared to South American countries. The peak season for the avocado is from October to April, but it is strongly present all year round on the shelves.

There are several varieties of avocados in Morocco, but eight varieties dominate the market: Ettinger, Fuerte, Hass, Reed, Zutano, Bacon, Maluma Hass and Choquette. Despite this importance and the different properties of avocado, the study of these different varieties in Morocco has not yet been approached. Moreover, such an investigation seemed to us to be important. In this context that our work is focused. Indeed, the objective of this work was a global investigation about mineral, phytochemical, and nutritional composition of different avocado as well as the evaluation of their antioxidative properties.

## Material and methods

### Plant material, solvent and reagent

**Figure 1:**
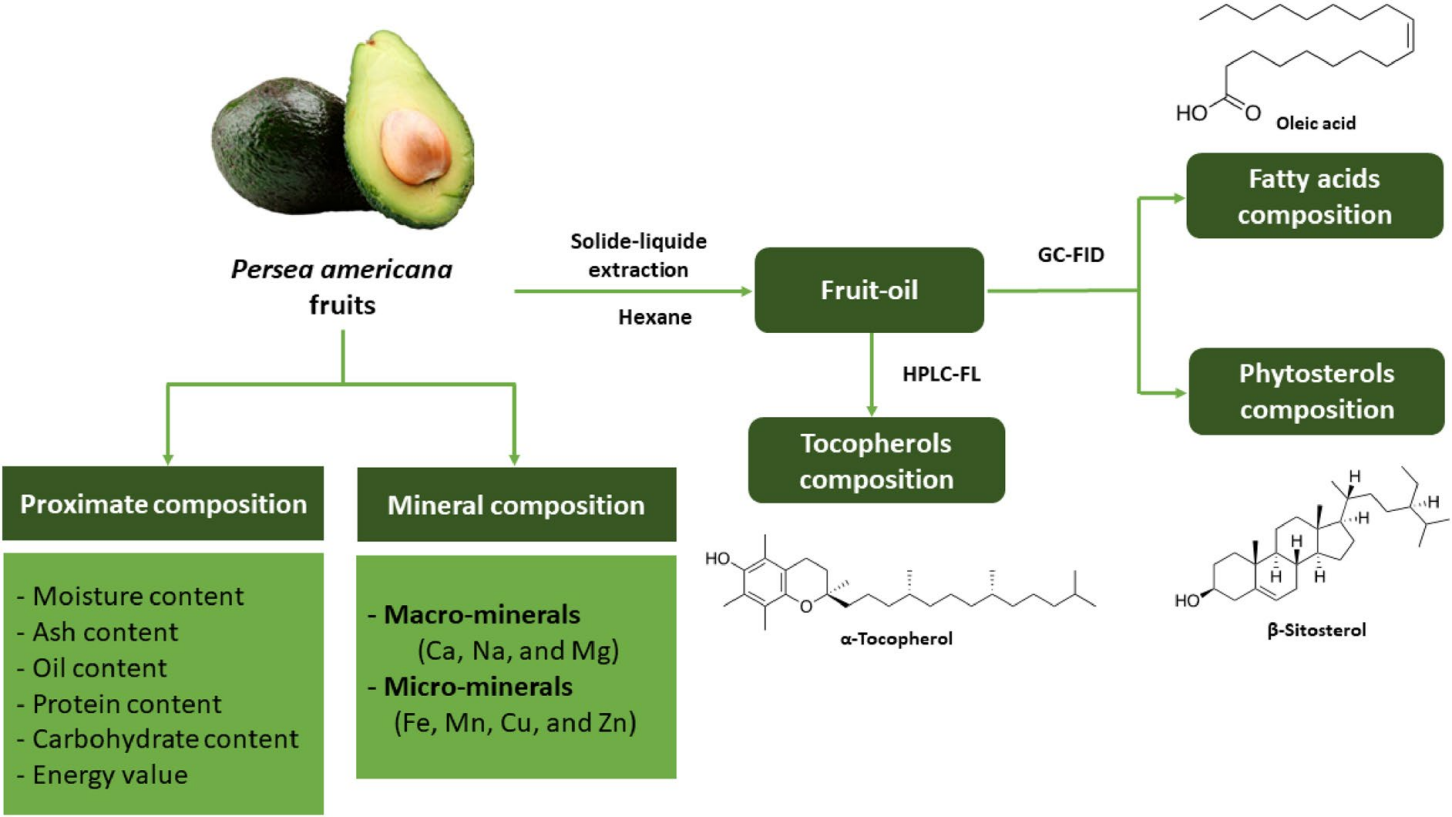
Summary of methodologies.

Avocado fruits used in this study were harvested during November and December 2022 from Rabat-Sale-Kenitra region (34°1′15.1752'' N, 6°50′29.9400'' W), Morocco. The pulps of the eight studied varieties (Ettinger, Fuerte, Hass, Reed, Zutano, Bacon, Maluma Hass, and Choquette) were dried in the laboratory using a ventilated oven. Once dried, the pulps were weighed, milled into fine powder using a blender, and then stored in a refrigerator at −4 °C until use. The flow of the conducted research is summarized in Fig. 1.

All reagents and solvents used were of analytical grade, except for the mobile phase used for HPLC, which was of chromatographic grade, and purchased from Professional Labo, Casablanca, Morocco.

### Proximate composition

Avocado pulps powder was used for proximate composition determination. All the tests were performed in triplicate. The moisture content (MC) was determined according to ISO 665:2020 method^11^. About 5 g of pulp from each avocado variety was weighed and dried in a ventilated oven at 106 °C, until a constant weight was reached. The ash content (AC) of pulps powder was determined following ISO 18122: 2022 method^12^. The crucibles used were oven dried at 105 °C for one hour, then cooled in the desiccator to room temperature and weighed. About 5 g of pulp powder were weighed into crucibles and the whole set was put into a muffle oven where they were heated gradually. After about 1 h of heating from 0 to 250 °C, the temperature was raised to 600 °C and burning continued for another 6 h. The crucibles were removed and allowed to cool in a desiccator to room temperature before weighing. The percentage of total ash was calculated. The oil contents (OC)were carried out according to ISO 659:2009 method^13^. About 30 g of powder from each dry pulp sample was extracted by Soxhlet apparatus for 8 h, using pure hexane as solvent. The solvent was removed under reduced pressure using a vacuum a rotary evaporator and the oil was stored at −4 °C until further analysis. The extraction yield was calculated gravimetrically. The Kjeldahl method was used for the determination of the total nitrogen content^14^. The samples were digested with a mixture of H_2_SO_4_, Se and C_7_H_6_O_3_. The resulting mixture was digested for 5 h at 300 °C by using a digestion block (QBlock series, Ontario, Canada). Then the mixture was filtered and treated with 5.5 mL of the buffer solution (4 mL of Na_2_[Fe (CN)_5_NO] and 2 mL of NaClO). The mixture was kept in the dark for 15 min at 37 °C, then the absorbance of each sample was measured at 650 nm. Protein content (PC) was determined by multiplying the nitrogen content by the conversion factor of 6.25.

Carbohydrate content (CC), and energy value (EV) were determined from protein content, moisture content, ash content, and oil content using the following equations:

$$ {\text{Carbohydrate content }}\left( {{\text{g}}/{1}00{\text{g}}} \right) \, = {1}00 - \, \left( {{\text{MC}} + {\text{ PC }} + {\text{AC}} + {\text{OC}}} \right) $$

$$ {\text{Energy value }}\left( {{\text{kCal}}/{1}00{\text{g}}} \right) \, = \, \left( {{8}.{37} \times {\text{OC}}} \right) \, + \left( {{4}.{2} \times {\text{CC}}} \right) \, + \left( {{2}.{62} \times {\text{ PC}}} \right) $$

### Mineral determination

The concentrations of metallic elements are determined after degreasing according to the method described by Munter and Grande (1981)^15^**.** About 500 mg of each sample were weighed into a glass tube and dissolved in 6 mL of 70% concentrated HNO_3_. The tubes were then placed in a digestion block (QBlock series, Ontario, Canada) and the whole was heated for 60 min at 90 °C. Then 3 mL of H_2_O_2_ (30%) were added to each tube and the mixture was heated at 90 °C for 15 min, then 3 mL of HCl (6 M) were added. The mineral solution in each tube was cooled and filtered and the solution was adjusted to volume of 10 mL. The concentrations of calcium, copper, iron, magnesium, manganese, sodium, and zinc were determined by inductivity coupled plasma-optical emission spectrometry (ICP-OES); (ICAP-7000 Duo, Thermo Fisher Scientific, France).

### Fatty acids composition

The fatty acids composition was assessed, after transesterification to their methyl esters derivatives (FAMEs) according to ISO 12966-2:2017 and ISO 12966-4:2015^16,17^ methods. The FAMEs analysis was carried out with an Agilent HP5890 gas chromatography system equipped with a flame ionization detector (FID), and a CP-Sil 88 capillary column (100 m × 250 µm i.d, 0.2 µm film thickness). The carrier gas was nitrogen, and the flow rate was 1 mL/min. The injector and detector were operated at 250 °C. The initial oven temperature was set at 155 °C for 5 min, increased to 230 °C at a rate of 1.5 °C/min, and maintained for 10 min isotherm. The injection volume was 1 µL in a split mode (1:50). The peak areas were computed by the integration software, and percentages of FAMEs were obtained by direct internal normalization as weight percentage. The standard mixture of fatty acid methyl esters (Sigma Chemical Co.) was used for the identification of the peaks.

### Phytosterols composition

Phytosterol composition and content were performed according to ISO 12228-1:2014 method^18^. After a trimethylsilylation of the crude sterol fraction, the composition was carried out using an Agilent 6890 gas chromatography system equipped with a SE 54 CB column capillary column (50 m × 320 µm i.d., 0.25 µm film thickness), and coupled to flame ionization detector. The carrier gas used was helium (flow rate: 1.6 mL/min). The injector and detector temperature was 320 °C. The oven temperature was programmed from 245 to 260 °C (5 °C/min). The injection volume was 1 µL in a split mode (1:20). The results were expressed as the relative percentage of the area of each individual sterol peak to the area of all sterol peaks. Cholestan-3-ol was used as an internal standard.

### Tocopherols composition

Tocopherol content and composition was determined using high-performance liquid chromatography according to ISO 9936:2016 method^19^. About, 20 µL of the filtrated solution (150 mg/mL of oil in n-heptane) was directly injected into a Merck-Hitachi low-pressure gradient system equipped with a Diol phase HPLC column (25 cm × 4.6 mm i.d.), a L-6000 pump, a fluorescence spectrophotometer (Merck-Hitachi F-1000), and a ChemStation integration system. The mobile phase was composed of a mixture of n-heptane and tert-butyl methyl ether (99:1, V:V) with a flow rate of 1.3 mL/min. The fluorimeter excitation and emission wavelengths were 290 nm and 317 nm, respectively. Identification of tocopherols was based on the comparison of retention time with reference standards and quantification was carried out using α-tocopherol as external standards.

### Statistical analysis of results

Summary data was reported as mean values ± standard deviation. Associations between tested quality traits were assessed using the Pearson correlation coefficient (r) with the metan package. ANOVA test was used for the analysis of variance. Tukey’s post-hoc test was applied to compare the differences between the mean values. Principal component analysis (PCA) was performed using the Factoextra and FactoMineR packages. All statistical analysis was conducted using RStudio version 1. 3.1093.

### Plant protection statement

We complied with international guidelines for biodiversity and bioethics, specifically, the Convention on Biological Diversity (CBD) and the Nagoya Protocol on Access and Benefit-sharing. This ensures that all plant materials were collected in a way that respects the sovereignty rights of countries and Indigenous communities over their biological resources. We ensured that no plant species classified as endangered or threatened were unduly affected by our research activities. Where necessary, special care was taken to minimise impact on such species and their habitats. We declare that this study did not involve any genetically modified organisms (GMOs) and that all necessary precautions were taken to prevent any unintended release or dissemination of plant species into inappropriate environments. Furthermore, we confirm that we have followed best practices for the storage, transport, and disposal of plant materials.

The authors of this study are committed to the highest standards of research integrity and ethics, respecting biodiversity, ecosystems, and the rights of local and Indigenous communities.

## Results and discussion

### Proximate composition

The assessment of the proximate composition and the nutritional value allowed a better knowledge for the farmers' perceptions of the avocado varieties in Morocco. A summary of the obtained results is illustrated in Table 1. The analysis of variance of proximate composition revealed a significant (*p* < 0.05) difference between tested varieties (Table 1). In this study, moisture content ranged from 57.88 g/100 g to 84.71 g/100 g and decreased as the oil content increased. Our results showed that the variety Maluma Hass had the highest moisture content value (84.71 ± 0.15 g/100 g), while its oil content was very low (8.41 ± 0.04 g/100 g). Whereas Ettinger had the lowest moisture content (57.88 ± 0.50 g/100 g), with the highest oil content (30.44 ± 0.04 g/100 g). Moisture content among tested varieties was relatively high which is in agreement with similar reported findings^20–22^. Moisture content constituted major part of the pulp. Many studies reported that avocado fruit is very susceptible to fungal spoilage due to the high level of moisture content^23^. Thus, low moisture content (50 g/100 g to 60 g/100 g) could delay the growth of spoilage microorganisms and enhance shelf life of avocado fruits^24^. Our outcomes revealed that oil content varied from 8.41 g/100 g (Ettinger) to 30.44 g/100 g (Maluma Hass). Oil content in Reed and Ettinger was higher than values reported by previous studies^20^. Avocado pulps ash values varied from 0.57 g/100 g to 1.37 g/100 g, with a mean of 1.01 g/100 g. Similarly, Nnaji et al. (2016)^20^, reported similar mean value that was 1.04 g/100 g. in contrast, Bora et al.,^25^ reported a low mean ash content in avocado pulps.

**Table 1:** Proximate composition (moisture content, oil content, ash content, protein content, and total carbohydrates), and energy value of avocado pulps.

	Bacon	Choquette	Ettinger	Fuerte	Hass	Maluma Hass	Reed	Zutano
MC (g/100 g)	75.39 ± 0.06^c^	75.74 ± 0.03^c^	57.88 ± 0.50^f^	69.21 ± 0.41^d^	76.03 ± 0.41^c^	84.71 ± 0.15^a^	67.01 ± 0.10^e^	78.45 ± 0.14^b^
OC (g/100 g)	13.69 ± 0.10^d,e^	13.33 ± 0.02^e^	30.44 ± 0.04^a^	13.93 ± 0.29^d^	13.45 ± 0.14^d,e^	8.41 ± 0.04^f^	18.83 ± 0.09^b^	14.65 ± 0.05^c^
AC (g/100 g)	0.88 ± 0.00^d,e^	1.24 ± 0.0^b^	1.12 ± 0.08^b,c^	1.11 ± 0.04^c^	0.99 ± 0.01^c,d^	0.82 ± 0.02^e^	1.37 ± 0.04^a^	0.57 ± 0.02^f^
PC (g/100 g)	8.61 ± 0.06^a^	8.17 ± 0.03^d^	8.3 ± 0.02^c^	7.68 ± 0.06^e^	8.47 ± 0.03^b^	6.27 ± 0.06^f^	8.18 ± 0.05^d^	5.78 ± 0.07^g^
TC (g/100 g )	9.1 ± 0.04^d^	8.8 ± 0.07^e^	9.49 ± 0.0^c^	14.61 ± 0.03^a^	8.87 ± 0.02^e^	5.63 ± 0.05^g^	11.88 ± 0.06^b^	5.93 ± 0.04^f^
E (kcal/100 g)	163.3 ± 0.07^d^	158.7 ± 0.04^e^	316.8 ± 0.06^a^	187.5 ± 0.05^c^	158 ± 0.06^f^	99.9 ± 0.04^h^	219.3 ± 0.06^b^	157.2 ± 0.06^g^

There is a significant difference between values in the same row that are followed by different letters (*p* < 0.05), according to the Tukey test. Each value is a mean of the results obtained on 2 trials ± standard deviation of this mean.

*MC* moisture content, *OC* oil content, *AC* ash content, *PC* protein content, *TC* total carbohydrates, *E* energy value.

The crude protein content (N × 6.25) of the fruit pulps varied from 5.78 g/100 g to 8.61 g/100 g, Bacon had the highest level, while Zutano showed the lowest value. Interestingly, protein content level in tested varieties was higher than previous fundings^20,26–34^ . The total carbohydrate content ranged from 5.63 g/100 g to 14.61 g/100 g, among the studied varieties. Maluma Hass had the lowest value, while Fuerte showed the highest carbohydrate content, with a value of 14.61 ± 0.03 g/100 g. In a 100-g of avocado, the number of kilocalories ranged between 99.9 kcal/100 g (Maluma Hass) and 316.8 kcal/100 g (Ettinger), according to the studied varieties. The high energy value of avocado pulps with a low carbohydrate content suggests their use for weight loss.

The ash contents of all studied samples were quite similar to those reported by Nwaokobia et al.,^35^ (with a value of 1.02 g/100 g), in contrast the moisture, carbohydrate, and oil contents were different. Other studies have shown that avocado pulp contained between 0.45 g/100 g and 1.77 g/100 g of ash content, moisture content between 67.87 g/100 g and 83.59 g/100 g, carbohydrates between 1.9 g/100 g and 8.64 g/100 g, lipids content between 6.53 g/100 g and 23.5 g/100 g, and protein between 1.20 g/100 g and 2.76 g/100 g^20,26–32,34^.

### Minerals determination

Minerals are required for the proper functioning of the body. Furthermore, they play an important role in protecting the immune system against infection, inflammation, and even cancer^36^. In this study, the concentrations of three macroelements (Ca, Mg, and Na), and four microelements (Fe, Zn, Cu, and Mn) were investigated. The obtained results (Table 2) showed significant difference (*p* < 0.05) of the mineral profile among the studied varieties. Sodium was the most dominant macro-mineral, with Maluma Hass having the highest value (12,056.19 ± 3.29 mg/kg). In addition, calcium concentration was significantly high in avocado pulp, the range was from 295.95 mg/kg to 531.67 mg/kg, with Ettinger variety showing the highest concentration, whereas Hass variety contained the lowest concentration. Calcium plays an essential role for the formation of blood clots, maintaining blood pressure, as well as contracting muscles^37,38^. As for magnesium, which was the least present macroelements, Reed variety showed the highest concentration (339.84 ± 0.22 mg/kg), whereas Zutano and Hass varieties showed slightly lower concentration 246.29 ± 1.38 mg/kg, and 247.09 ± 0.12 mg/kg. As a result, the avocado fruit can be recommended to those suffering from stress, fatigue, and anxiety.

**Table 2:** Mineral profile of the eight varieties of Moroccan avocado pulp (mg/kg).

	Bacon	Choquette	Ettinger	Fuerte	Hass	Maluma Hass	Reed	Zutano
Ca	404.17 ± 0.36^b^	302.85 ± 1.10^g^	531.67 ± 1.03^a^	356.67 ± 1.86^d^	295.95 ± 0.63^h^	312.63 ± 0.26 ^f^	366.52 ± 1.90^c^	327.04 ± 0.30^e^
Cu	86.68 ± 0.01^e^	90.38 ± 0.45^d^	112.31 ± 0.19^a^	85.92 ± 0.43^e^	101.04 ± 0.07^b^	96.34 ± 0.12^c^	82.85 ± 0.65^f^	91.45 ± 1.72^d^
Fe	12.07 ± 0.09 ^b.c^	12.08 ± 0.04 ^b.c^	11.6 ± 0.06^c^	8.23 ± 0.08^e^	20.32 ± 0.25^a^	10.4 ± 0.13^d^	12.63 ± 0.18^b^	12.61 ± 0.04^b^
Mg	304.98 ± 0.06^b^	289.87 ± 0.04^c^	288.49 ± 0.34^c^	274.74 ± 0.26^d^	247.09 ± 0.12^f^	261.16 ± 0.87^e^	339.84 ± 0.22^a^	246.29 ± 1.38 ^f^
Mn	18.45 ± 0.02^a^	7.48 ± 0.04^g^	12.39 ± 0.09^c^	9.35 ± 0.05 ^f^	14.02 ± 0.03^b^	10.16 ± 0.08^e^	11.97 ± 0.03^d^	12.6 ± 0.08^c^
Na	10,947.21 ± 0.01^b^	7235.52 ± 0.19^e^	5783.01 ± 0.53^f^	7173.04 ± 0.06^e^	8396.5 ± 0.28^d^	12,056.19 ± 3.29^a^	10,894.89 ± 0.6^b^	10,466.29 ± 85.09^c^
Zn	2.93 ± 0.01^e^	3.71 ± 0.00^c^	3.4 ± 0.00^d^	5.66 ± 0.03^a^	4.02 ± 0.01^b^	3.7 ± 0.00^c^	1.72 ± 0.00^g^	2.82 ± 0.00 ^f^

There is a significant difference between values in the same row that are followed by different letters (*p* < 0.05), according to the Tukey test. Each value is a mean of the results obtained on 2 trials ± standard deviation of this mean.

*Ca* calcium, *Cu* copper, *Fe* iron, *Mg* magnesium, *Mn* manganese, *Na* sodium, *Zn* zinc.

Although microelements are present in small amounts, they are essential for all living organisms, including plants, and are involved in many physiological functions^39^**.**

Copper was found to have the highest concentration among microelements, with the highest content recorded in Ettinger variety (112.31 ± 0.19 mg/kg), while Bacon (86.68 ± 0.01 mg/kg), and Fuerte (85.92 ± 0.43 mg/kg) had the lowest contents. Several physiological processes rely on copper, including angiogenesis, neurohormone homeostasis, brain development, pigmentation, and immune function^40^. Iron, and Manganese were recorded almost with similar proportions. The Hass variety had the highest iron content (20.32 ± 0.25 mg/kg), while Fuerte variety showed the lowest value (8.23 ± 0.08 mg/kg). The variety Bacon had the highest Manganese content with a value of 18.45 ± 0.02 mg/kg, while the Choquette had only 7.48 ± 0,04 mg/kg. In contrast, Zinc was the underrepresented microelement, the highest concentration (5.66 ± 0.03 mg/kg) was found in the Fuerte variety, while Reed had only 1.72 ± 0.00 mg/kg.

Many studies have indicated that avocado pulp contains mineral content ranging from 17.4 mg/kg to 150 mg/kg of calcium, 1.6 mg/kg to 1.8 mg/kg of copper, and 5 mg/kg to 20 mg/kg of Iron. The magnesium content ranges from 14 mg/kg to 600 mg/kg, the manganese content ranges from 0.3 mg/kg to 27.3 mg/kg, the sodium content ranges from 4.7 mg/kg to 150 mg/kg, and the zinc content ranges from 0.5 mg/kg to 6.8 mg/kg^28,31,34,41^.

### Chemical composition of the vegetable oil of the avocado

Fatty acid composition.

Fatty acid composition was assessed after conversion to their methyl esters derivatives and analysis by GC-FID. fatty acid composition of avocado pulp oils is displayed in Table 3. The obtained results indicated a significant difference between the eight studied varieties (*p* < 0.05).

**Table 3:** Fatty acid composition of avocado oil.

	Bacon	Choquette	Ettinger	Fuerte	Hass	Maluma Hass	Reed	Zutano
C16:0	15.34 ± 0.01 ^f^	18.19 ± 0.01^d^	15.23 ± 0.13^f^	15.67 ± 0.06^e^	20.94 ± 0.05^b^	22.42 ± 0.01^a^	18.43 ± 0.01^c^	14.44 ± 0.02^g^
C16:1	5.76 ± 0.01^e^	4.42 ± 0.01^f^	8.6 ± 0.11^c^	2.06 ± 0.01^g^	9.83 ± 0.04^a^	9.52 ± 0.01^b^	7.55 ± 0.01^d^	4.47 ± 0.0^f^
C18:0	0.59 ± 0.01^b^	0.6 ± 0.01^b^	0.4 ± 0.11^b^	0.85 ± 0.04^a^	0.44 ± 0.08^b^	0.49 ± 0.0^b^	0.53 ± 0.03^b^	0.59 ± 0.01^b^
C18:1	67.79 ± 0.01^b^	62.34 ± 0.01^c^	60.71 ± 0.05^e^	57.48 ± 0.06 ^f^	54.51 ± 0.12^g^	50.38 ± 0.01^h^	61.18 ± 0.09^d^	71.49 ± 0.01^a^
C18:2	9.51 ± 0.01^g^	12.51 ± 0.01^e^	13.4 ± 0.01^c^	19.81 ± 0.04^a^	12.96 ± 0.04^d^	15.17 ± 0.01^b^	10.59 ± 0.01^f^	7.79 ± 0.01^h^
C18:3	0.54 ± 0.01^f^	1.42 ± 0.01^b^	1.06 ± 0.06^c^	1.62 ± 0.07^a^	0.88 ± 0.04^d^	1.55 ± 0.01^a,b^	0.8 ± 0.01^d,e^	0.69 ± 0.01^e,f^
C20:1	0.22 ± 0.01^c^	0.29 ± 0.02^a^	0.23 ± 0.01^b,c^	0.26 ± 0.02^a,b,c^	0.17 ± 0.01^d^	0.27 ± 0.01^a,b^	0.23 ± 0.01^b,c^	0.26 ± 0.01^a,b,c^

Based on Tukey's test, the means across each row with different superscript letters differ significantly (*p* < 0.05). Each value is a mean of the results obtained on 2 trials ± standard deviation of this mean.

C16:0: palmitic acid; C16:1: palmitoleic acid; C18:0: stearic acid; C18:1: oleic acid; C18:2: oleic acid; C18:3: linolenic acid; C20:1: gadoleic acid.

**Figure 2:**
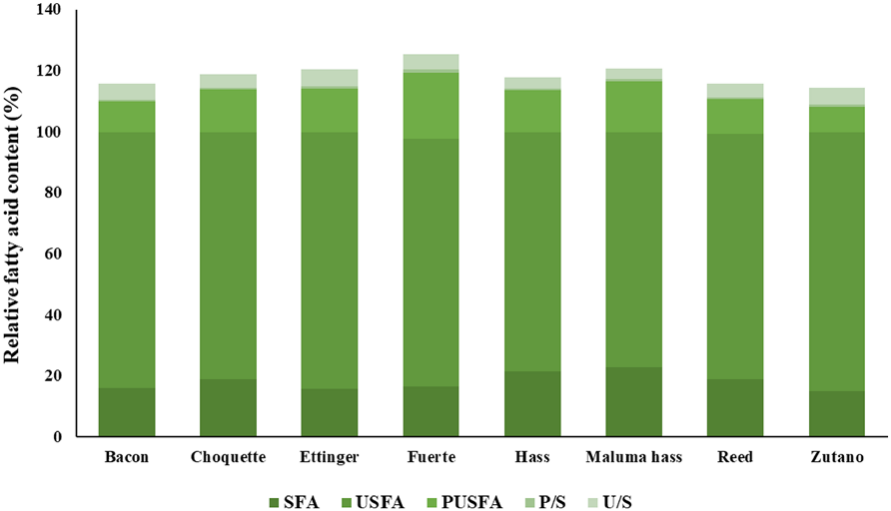
Total fatty acid content (%) of different varieties of avocado fruit.

The avocado pulps contained a significant amount of oil, which makes it an important source for food and cosmetic industries^42^. Figure 2 illustrates the total levels of unsaturated fatty acids (USFAs), saturated fatty acids (SFAs), and polyunsaturated fatty acids (PUFAs), as well as the P/S and U/S ratios. Unsaturated fatty acids (USFAs) accounted for 76.89 g/100 g to 84.7 g/100 g of total fatty acids, in which 8.48 g/100 g to 21.43 g/100 g were polyunsaturated fatty acids, while 15.03 g/100 g to 22.91 g/100 g was monounsaturated (MUFA). The avocado pulp oil contained five unsaturated fatty acids and two saturated fatty acids. Among the seven identified fatty acids, oleic acid (C18:1) was the predominant fatty acid, its rate varied from 50.38 g/100 g (Maluma Hass) to 71.49 g/100 g (Zutano). Avocado oil can thus be regarded as an oleic oil. It has been suggested that oleic acid may have a moderate and controversial positive effect on LDL cholesterol^43,44^. Linoleic acid (C18:2) was also detected with a significant level varying from 7.79 g/100 g (Zutano), to 19.81 g/100 g (Fuerte). Two other UFAs were also identified, which are linolenic acid (C18:3) (0.54 g/100 g Bacon to 1.62 g/100 g Fuerte), and palmitoleic acid (C16:1) (2.06 g/100 g Choquette to 9.83 g/100 g Hass). Palmitic acid (C16:0) was the main saturated fatty acid, with a value ranging from 14.44 g/100 g to 22.42 g/100 g, the variety with the highest level was Maluma Hass, whereas Zutano presented the lowest value. In addition, stearic and arachidic acids were found in trace amounts (less than 1 g/100 g of total fatty acids) in avocado fruit pulp oils, with their contents ranging from 0.4 g/100 g to 0.85 g/100 g and 0.17 g/100 g to 0.29 g/100 g respectively.

Similarly, Dubois et al.^45^ found that avocado vegetable oil contains over 60 g/100 g of monounsaturated fatty acids, oleic acid was the foremost detected fatty acid (60.3 g/100 g). In contrast to our findings, they found a higher percentage of saturated fatty acids (16.4%). The main saturated fatty acid identified by Dubois et al.^45^ was palmitic acid (15.7 g/100 g). There was a significant amount of polyunsaturated fatty acids (15.2 g/100 g), linoleic acid was the major compound (13.7 g/100 g). The PUFA/PFA ratio in avocado oil was higher than that in olive oil, according to Berasategi et al*.*^46^. Jorge et al.^47^ reported that avocado oil from two varieties (Margarida and Hass) contained significant amounts of oleic acid, that was 54.72 g/100 g for the Hass variety, similarly to the same variety studied in our research, and 57.33 g/100 g for the Margarida variety, which is similar to the Fuerte variety in our study. In both varieties, Margarida and Hass, palmitic acid was 23.28 g/100 g and 19.43 g/100 g, respectively, while linoleic acid was 14.84 g/100 g and 13.22 g/100 g.

### Phytosterol composition

Total phytosterols content and composition were assessed by gas chromatography equipped with a flame ionization detector. Six phytosterols were identified in all studied samples (Table 4). The obtained results indicated a significant difference of phytosterols contents and compositions among the eight studied avocado varieties (*p* < 0.05). Total phytosterol contents ranged from 2818.86 mg/kg to 5401.36 mg/kg, Choquette (5401.36 ± 0.08 mg/kg), Fuerte (5378.86 ± 0.09 mg/kg), and Maluma Hass (5303.95 ± 0.06 mg/kg) had the highest values, while Maluma Hass recorded the lowest phytosterol content (2818.86 ± 0.08 mg/kg). In all varieties, β-sitosterol was the major detected phytosterol with a value ranging from 2365.58 ± 1.26 mg/kg (Bacon) to 4559.27 ± 2.31 mg/kg (Maluma Hass). Followed by Campesterol (113.46 mg/kg Bacon to 372.97 mg/kg Choquette), and Δ-5-avenasterol (145.38 mg/kg Reed to 328.92 mg/kg Fuerte). Traces of cholesterol and stigmasterol were observed in the different varieties at levels below 1%. β-Sitosterol has interesting beneficial and physiological effects on health, according to bouic^48^, it plays an important role in immunity system, treatment of cancer, HIV, and infections.

**Table 4:** Phytosterol contents and compositions of oils from eight different avocado varieties.

	Bacon	Choquette	Ettinger	Fuerte	Hass	Maluma Hass	Reed	Zutano
CholS	18.75 ± 10.56^a,b^	12.96 ± 2.29^a,b^	17.93 ± 2.31^a,b^	6.73 ± 1.14^b^	6.34 ± 3.4^b^	10.61 ± 1.5^a,b^	10.7 ± 3.03^a,b^	26.4 ± 2.47^a^
CampS	113.46 ± 1.8^h^	372.97 ± 2.68^a^	155.66 ± 1.15^f^	303.91 ± 4.56^b^	244.71 ± 0.62^e^	287.74 ± 1.88^c^	263.11 ± 3.79^d^	133.28 ± 1.01^g^
StigS	25.23 ± 1.8^b^	29.17 ± 2.29^b^	20.37 ± 1.61^b,c^	40.88 ± 5.33^a^	26 ± 1.54^b^	20.95 ± 1.88^b,c^	3.03 ± 0.25^d^	12.47 ± 1.23^c,d^
β-SitoS	2365.58 ± 1.26^h^	4541.46 ± 1.6^b^	2687.6 ± 2.02^f^	4502.1 ± 6.01^c^	3650.05 ± 3.64^d^	4559.27 ± 2.31^a^	2997.12 ± 1.43^e^	2418.52 ± 2.34^g^
Δ5-AvenS	145.46 ± 2^f^	229.29 ± 2.68^e^	265.85 ± 2.07^c^	328.92 ± 2.67^a^	309.6 ± 4.02^b^	242.13 ± 3.37^d^	145.38 ± 3.79^f^	156.19 ± 0.59^f^
TS	2818.86 ± 0.08^h^	5401.36 ± 0.08^a^	3259.87 ± 0.07^f^	5378.86 ± 0.09^b^	4369.74 ± 0.08^d^	5303.95 ± 0.06^c^	3567.58 ± 0.11^e^	2900.42 ± 0.4^g^

Based on Tukey's test, the means across each row with different superscript letters differ significantly (*p* < 0.05). Each value is a mean of the results obtained on 2 trials ± standard deviation of this mean.

*CholS* cholesterol, *CampS* campesterol, *StigS* stigmasterol, *β-SitoS* β-sitosterol, *Δ5-AvenS* Δ5-avenasterol, *TS* total sterol content.

Compared to other studies, Salgado et al.^49^ reported a lower β-sitosterol content, representing 71.8% of total sterols. Our results were in agreement to those reported by Santos et al.^50^, which indicated similar contents of β-sitosterol (87.6%), campesterol (12.41%), and stigmasterol (0.04%). In addition, Berasategi et al.^46^ reported a lower campesterol level (180 mg/kg) compared to Choquette, Fuerte, Hass’, Maluma Hass, and Reed varieties, but higher to those of Bacon, Ettinger, and Zutano varieties. Berasategi et al.^46^ indicated also a lower Δ-5-avenasterol content (94 mg/kg), and a higher stigmasterol content (11 mg/kg).

### Tocopherol composition

The tocopherol content and composition of avocado oils were determined using a high-performance liquid chromatography coupled to a fluorescence detector. The obtained results revealed the presence of three tocopherols, namely α, β, and γ-tocopherols (Table 5). A significant difference was observed between the analyzed oils of the eight avocado varieties (*p* < 0.05). Tocopherols are known as the main antioxidant compounds, and contributes to the oil's stability^51^. Total tocopherol varied from 77.22 mg/kg to 332.18 mg/kg. Fuerte variety had the highest level (332.18 ± 0.04 mg/kg) while Bacon had the lowest value (77.22 ± 0.01 mg/kg). α-Tocopherol was the major tocopherol detected in all studied varieties, with values ranging from 30.08 ± 0.02 mg/kg (Bacon) to 182.94 ± 0.01 mg/kg (Maluma Hass). While, γ-tocopherol was detected in four varieties only, namely Choquette (52.33 ± 0.03 mg/kg), Maluma Hass (51.63 ± 0.01 mg/kg), Zutano (14.12 ± 0.01 mg/kg), and Bacon (7.87 ± 0.03 mg/kg). β-Tocopherol was only detected in Zutano with a value of 2.48 ± 0.01 mg/kg. Among the tocopherol homologs, the α-tocopherol has the highest biological potency, while γ-tocopherol was reported as the most potent antioxidant. Flores et al.(2014)^52^ reported similar tocopherol content as Ettinger (40.5 mg/kg), and Zutano (103.5 mg/kg) varieties. In contrast, Ramos-Aguilar et al.(2021)^53^ reported lower tocopherol contents than those found in our study for Choquette, Fuerte, Hass, Maluma Hass, and Zutano varieties cultivated in Mexico. Manaf et al.(2019)^54^ found four tocopherols in oil extracted from three Indonesian avocados, including α-tocopherol (40 mg/kg to 45 mg/kg), β-tocopherol (0.8 mg/kg to 2.0 mg/kg), γ-tocopherol (3.8 mg/kg to 4.2 mg/kg), and δ-tocopherol (0.04 mg/kg to 0.08 mg/kg). the reported α-tocopherol concentrations were similar to those found in the variety Ettinger, β-tocopherol contents were similar to those found in Zutano, while γ-tocopherol contents were lower than our values. On the other hand, δ-tocopherol was not detected in tested avocado varieties.

**Table 5:** Tocopherol contents and compositions of avocado oils from different avocado varieties.

	Bacon	Choquette	Ettinger	Fuerte	Hass	Maluma Hass	Reed	Zutano
α-Toco	30.08 ± 0.02^h^	89.91 ± 0.01^e^	46.84 ± 0.01^g^	177.9 ± 0.02^b^	159.8 ± 0.12^c^	182.94 ± 0.01^a^	79.07 ± 0.04 ^f^	98.39 ± 0.01^d^
β-Toco	ND	ND	ND	ND	ND	ND	ND	2.48 ± 0.01^a^
γ-Toco	7.87 ± 0.03^d^	52.33 ± 0.03^a^	ND	ND	ND	51.63 ± 0.01^b^	ND	14.12 ± 0.01^c^
T.Toco	77.22 ± 0.01^h^	220.26 ± 0.01^d^	113.1 ± 0.01^g^	332.18 ± 0.04^a^	252.92 ± 0.06^c^	327.78 ± 0.02^b^	186.16 ± 0.08^e^	176.11 ± 0.01^f^

Based on Tukey's test, the means across each row with different superscript letters differ significantly (*p* < 0.05). Each value is a mean of the results obtained on 2 trials ± standard deviation of this mean.

*ND* not detected, *α-Toco* α-tocopherol, *β-Toco* β-tocopherol, *γ-Toco* γ-tocopherol, *T.Toco* total tocopherols.

**Figure 3:**
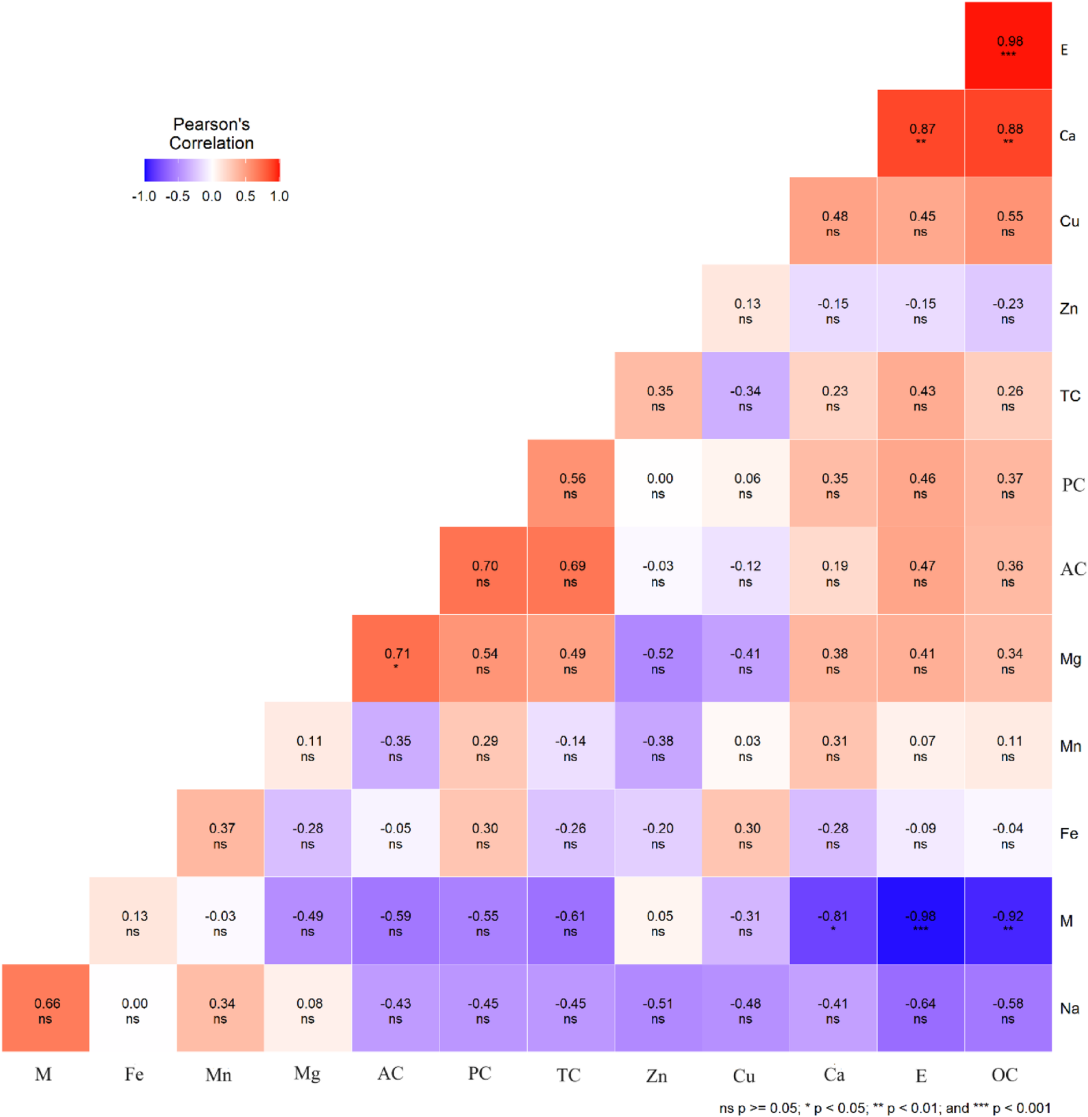
Correlation between proximate composition, energy value and micro and macroelements of eight varieties of avocado.

**Figure 4:**
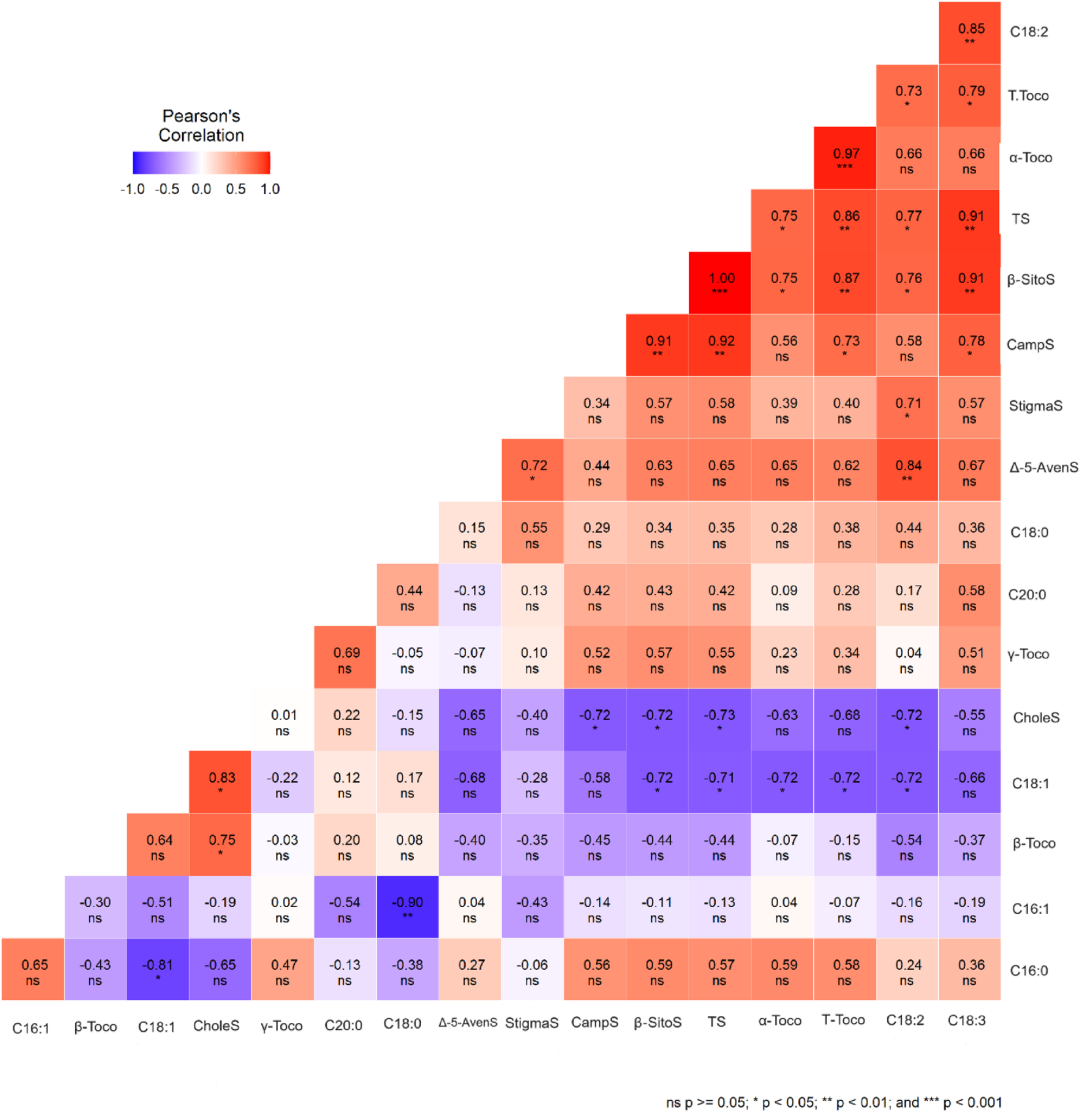
Correlation between fatty acids, sterols, and tocopherols.

The coefficient of correlation between the moisture, oil, ash, protein, carbohydrate content, energy value, and micro and macro elements in avocado pulp is presented in Fig. 3 and Supplementary Table 1. The energy value showed a significant positive correlation (r = 0.98, ***P < 0.001) with oil content and Calcium (r = 0.88, P < 0.01), whereas the energy value was negatively correlated with moisture content (-0.92, P < 00.001). Moisture content was negatively correlated with oil content (r = -0.92, P < 0.01) and Calcium (r = −0.8, P < 0.05). A significant positive correlation was obtained between Ash content and magnesium; the coefficient of correlation was r = 0.71 (P < 0.05). whereas the correlation between ash and the remaining nutrients was statistically non-significant (Fig. 4). Similarly, Protein content showed no significant correlation with other tested traits.

The correlation coefficients between fatty acids, sterols, and tocopherols present in avocado oils of different varieties are summarized in Supplementary Table 2. The correlation analysis, there are many significant negative and positive correlations. Only results with p < 0.05, p < 0.01 and p < 0.001 are discussed here. There was a positive correlation between C18:3 and C18:2 (r = 0.85, **P < 0.01), total sterols (r = 0.91, **P < 0.01) and β-sitosterol (r = 0.91, **P < 0.01), but there was a weak positive correlation between total tocopherols and campesterol (r = 0.79, *P < 0.05 and r = 0.78, *P < 0.05 respectively). The C18:2 content was positively correlated with Δ5-avenasterol (r = 0.84, **P < 0.01), total tocopherols (r = 0.73, *P < 0.05), total sterols (r = 0.77, *P < 0.05), β-sitosterol (r = 0.76, *P < 0.05) and stigmasterol (r = 0.71, *P < 0.05), whereas C18:2 content was negatively associated with cholesterol and C18:1 (r = -0.72, *P < 0.05 for both). A highly significant positive correlation was obtained between tocopherol content and α-tocopherol (r = 0.97, ***P < 0.001). Tocopherol content was also positively correlated with total sterols, β-sitosterol and campesterol (r = 0.86, **P < 0.01, r = 0.87, **P < 0.01, r = 0.73, *P < 0.05 respectively), whereasC18:2 was negatively correlated with C18:1(r = −0.72, *P < 0.05). α-Tocopherol showed a low positive correlation with total sterols and β-sitosterol (r = 0.75, *P < 0.05, for both), but a low negative correlation with C18:1(r = −0.72, *P < 0.05). A strong positive correlation among total sterols was observed with β-sitostérol (r = 1.00, ***P < 0,001), a positive correlation with campesterol (r = 0.92, **P < 0.01), and a weak negative correlation with cholesterol (r = −0.73, *P < 0.05) and C18:1(r = −0.71, *P < 0.05). β-Sitosterol had a positive correlation with campesterol (r = 0.91, **P < 0.01), but a weak negative correlation with cholesterol and C18:1(r = −0.72, *P < 0.05, for both). Campesterol and cholesterol were negatively correlated (r = −0.72, *P < 0.05). There was a weak positive correlation between stigmasterol and Δ5-avenasterol (r = 0.72, *P < 0.05). In addition, C18:0 showed a positive correlation with C16:1 (r = 0.90, **P < 0.01). On the other hand, Cholesterol showed a weak positive correlation with both C18:1 and α-tocopherol ((r = 0.83, *P < 0.05 and r = 0.75, *P < 0.05 respectively), while C18:1 showed a weak negative correlation with C16:0 (r = 0.81, *P < 0.05).

Correlation analysis was conducted to elucidate the associations among the assessed nutritional quality traits. This analysis provided insights into which parameters may be concurrently present when consuming these varieties.

### Principal component analysis (PCA)

**Figure 5:**
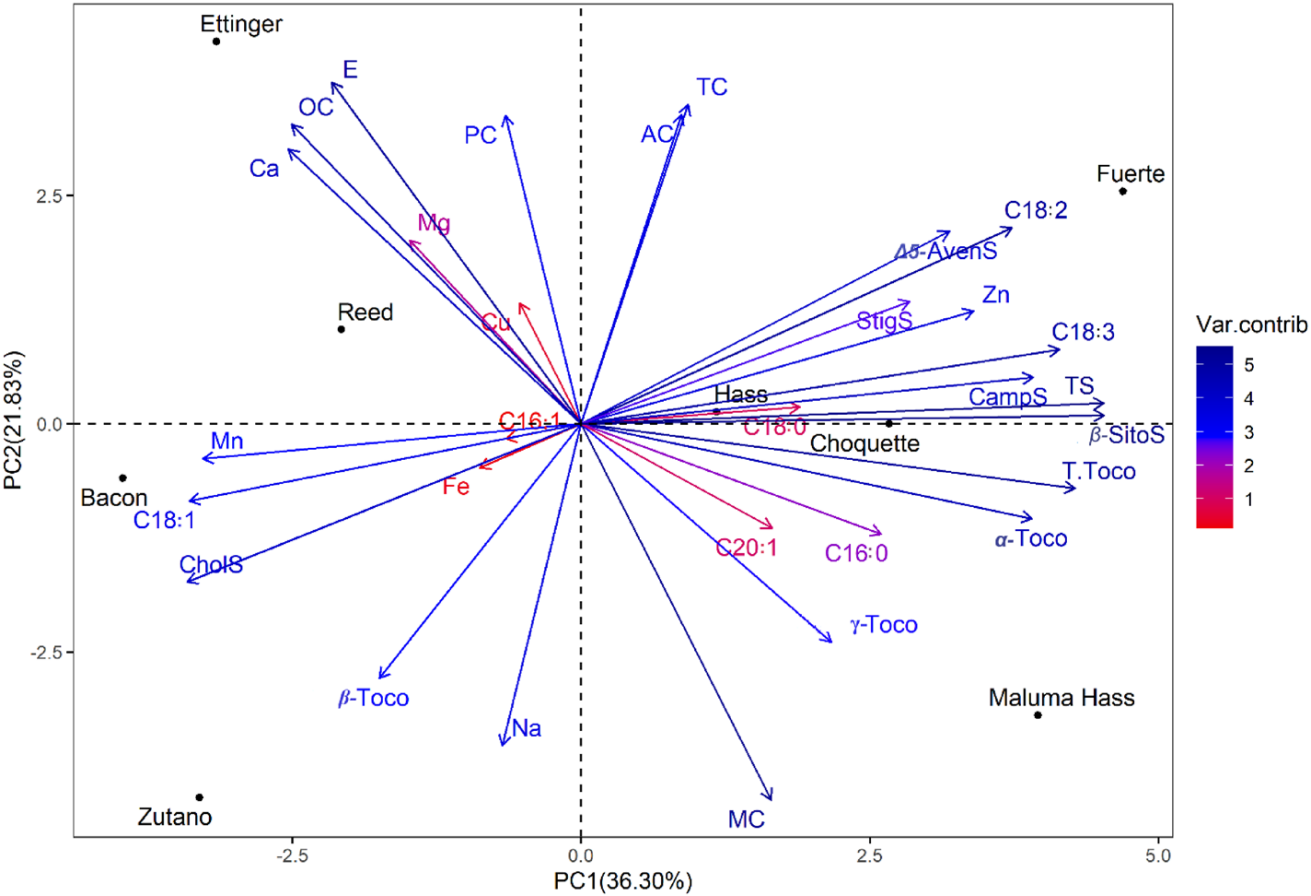
Principal component analysis and various quality traits contributing to the total variability. *Ca* calcium, *Cu* copper, *Fe* iron, *Mg* magnesium, *Mn* manganese, *Na* sodium, *Zn* zinc, *C16:0* palmitic acid, *C16:1* palmitoleic acid, *C18:0* stearic acid, *C18:1* oleic acid, *C18:2* oleic acid, *C18:3* linolenic acid, *C20:1* gadoleic acid, *CholS* cholesterol, *CampS* campesterol, *StigS* stigmasterol, *β-SitoS* β-sitosterol, *Δ5-AvenS* Δ5-avenasterol, *TS* total sterol content, *α-Toco* α-tocopherol, *β-Toco* β-tocopherol, *γ-Toco* γ-tocopherol, *T.Toco* total tocopherols.

Principal component analysis (PCA) was performed to study the relationships between quality parameters in the eight tested avocado varieties (Fig. 5). Eigenvalues of the studied traits and cumulative variances are reported for parameters in Table 6. The analysis revealed that the first three PCAs explained 73.02% of the total variability. PC1 and PC2 accounted for 36.30% and 21.83% of the total variation, while PC3 described 14.89% of the total variability. Total tocopherols, α-tocopherols, oleic Acid (C18:1), linoleic acid (C18:2), linolenic acid (C18:3), β‑sitosterol, campesterol, cholesterol, total sterol, zinc and Manganese revealed the highest contribution to the first principal component (PC1), while sodium, protein content, moisture content, total carbohydrates, energy value, ash content and oil content were the most contributing traits to the second principal component (PC2). PC3 variation was mainly due to iron, palmitic Acid (C16:0), palmitoleic Acid (C16:1) and stearic Acid (C18:0).

**Table 6:** Eigenvalues and eigenvectors of the three PCA dimensions of tested parameters in eight avocado varieties.

	PC1	PC2	PC3
Ca	2.77	6.49	0.09
Cu	0.12	1.25	6.70
Fe	0.33	0.17	**12.47**
Mg	0.95	2.90	0.63
Mn	**4.63**	0.11	1.48
Na	0.20	**8.92**	0.30
Zn	**4.99**	1.09	0.75
PC	0.19	**8.17**	1.32
MC	1.17	**12.18**	0.21
AC	0.33	**8.22**	0.06
TC	0.37	**8.77**	2.45
E	2.01	**10.03**	0.03
OC	2.71	**7.71**	0.03
α-Toco	**6.59**	0.78	0.69
β-Toco	1.32	5.58	2.92
γ-Toco	2.03	4.10	0.03
T.Toco	**7.90**	0.36	0.04
C16:0	2.91	1.05	**11.53**
C16:1	0.18	0.02	**19.91**
C18:0	1.55	0.02	**14.62**
C18:1	**4.96**	0.51	7.37
C18:2	**6.01**	3.31	0.10
C18:3	**7.40**	0.48	0.56
C20:1	1.18	0.93	10.22
β-SitoS	**8.85**	0.01	0.00
Δ5-AvenS	4.39	3.19	1.10
CampS	**6.62**	0.19	0.00
CholS	**5.02**	2.16	3.08
StigS	3.49	1.28	1.30
TS	**8.85**	0.04	0.00
Eigenvalue	10.89	6.55	4.47
Variance explained (%)	36.30	21.83	14.89
Total variance (%)	36.30	58.13	73.02

*Ca* calcium, *Cu* copper, *Fe* iron, *Mg* magnesium, *Mn* manganese, *Na* sodium, *Zn* zinc, *C16:0* palmitic acid, *C16:1* palmitoleic acid, *C18:0* stearic acid, *C18:1* oleic acid, *C18:2* oleic acid, *C18:3* linolenic acid, *C20:1* gadoleic acid, *CholS* cholesterol, *CampS* campesterol, *StigS* stigmasterol, *β-SitoS* β-sitosterol, *Δ5-AvenS* Δ5-avenasterol, *TS* total sterol content, *α-Toco* α-tocopherol, *β-Toco* β-tocopherol, *γ-Toco* γ-tocopherol, *T.Toco* total tocopherols.

Significant values are in bold.

## Conclusion

The current study investigated the nutritional value of eight varieties of avocados from Morocco. Our results revealed that avocado fruit pulps were rich in minerals (Ca, Cu, Fe, Mg, Mn, Na et Zn), protein and oil content. As a result of its high fat content, numerous properties and its many benefits for health, avocados are of great interest on the industrial level. The avocado oil is among the vegetable oils the easiest to extract. Among the fatty acids in the pulp, oleic acid (≥ 70%) is a major component, while the sterol fraction contains a high content of sterols and features β-sitosterol (≥ 80%) predominance. Analysis of the chemical composition of tocopherols indicates that α-Tocopherol is the major tocopherol. Based on the results of the present study, all avocados investigated, regardless of the variety, contain a variety of nutrients. Consequently, the pulp of these avocados needs to be valorized.

### Supplementary Information

Supplementary Table 1.

Supplementary Table 2.

## Data Availability

All data generated or analysed during this study are included in this published article.
