# Supplementary material for: Proximate composition, lipid and elemental profiling of eight varieties of avocado (Persea americana)
Source: Sci Rep. 2023 Dec 20;13:22767. doi: 10.1038/s41598-023-50119-y (PMC10733347; doi:10.1038/s41598-023-50119-y)
Supplement: Supplementary file 1 — Supplementary Table 1. [file 41598_2023_50119_MOESM1_ESM.docx]

Table 1. Correlation coefficients between micro and macroelements, proximate composition and energy value.

|  | Cu | Fe | Mg | Mn | Na | Zn | CP | M | Ash | TC | E | OC |
| --- | --- | --- | --- | --- | --- | --- | --- | --- | --- | --- | --- | --- |
| Ca | 0.48 | -0.29 | 0.38 | 0.31 | -0.41 | -0.15 | 0.35 | -0.81* | 0.19 | 0.23 | 0.87** | 0.88** |
| Cu | 1 | 0.30 | -0.41 | 0.03 | -0.48 | 0.13 | 0.06 | -0.31 | -0.12 | -0.34 | 0.45 | 0.55 |
| Fe |  | 1 | -0.28 | 0.37 | 0.00 | -0.20 | 0.30 | 0.13 | -0.05 | -0.26 | -0.09 | -0.04 |
| Mg |  |  | 1 | 0.11 | 0.08 | -0.52 | 0.54 | -0.49 | 0.71* | 0.49 | 0.41 | 0.34 |
| Mn |  |  |  | 1 | 0.34 | -0.38 | 0.29 | -0.03 | -0.35 | -0.14 | 0.07 | 0.11 |
| Na |  |  |  |  | 1 | -0.51 | -0.45 | 0.66 | -0.43 | -0.45 | -0.64 | -0.58 |
| Zn |  |  |  |  |  | 1 | 0.00 | 0.05 | -0.03 | 0.35 | -0.15 | -0.23 |
| CP |  |  |  |  |  |  | 1 | -0.55 | 0.70 | 0.56 | 0.46 | 0.37 |
| M |  |  |  |  |  |  |  | 1 | -0.59 | -0.61 | -0.98*** | -0.92** |
| Ash |  |  |  |  |  |  |  |  | 1 | 0.69 | 0.47 | 0.36 |
| TC |  |  |  |  |  |  |  |  |  | 1 | 0.43 | 0.26 |
| E |  |  |  |  |  |  |  |  |  |  | 1 | 0.98*** |

*^∗^P <* 0.05, *^∗∗^P <* 0.01 , *^∗∗∗^P <* 0.001 .
