# Supplementary material for: Proximate composition, lipid and elemental profiling of eight varieties of avocado (Persea americana)
Source: Sci Rep. 2023 Dec 20;13:22767. doi: 10.1038/s41598-023-50119-y (PMC10733347; doi:10.1038/s41598-023-50119-y)
Supplement: Supplementary file 2 — Supplementary Table 2. [file 41598_2023_50119_MOESM2_ESM.docx]

|  | β-Tocopherol | γ-Tocopherol | Total tocopherol | PA.C16.0 | PA.C16.1 | SA.C18.0 | OA.C18.1 | LA.C18.2 | LA..C18.3 | GA..C20.1 | β-sitosterol | Δ5-avenasterol | Campesterol | Cholesterol | Stigmasterol | Total Sterol content |
| --- | --- | --- | --- | --- | --- | --- | --- | --- | --- | --- | --- | --- | --- | --- | --- | --- |
| α-Tocopherol | -0.07 | 0.23 | 0.97*** | 0.59 | 0.04 | 0.28 | -0.72* | 0.66 | 0.66 | 0.09 | 0.75* | 0.65 | 0.56 | -0.63 | 0.39 | 0.75* |
| β-Tocopherol | 1 | -0.03 | -0.15 | -0.43 | -0.30 | 0.08 | 0.64 | -0.54 | -0.37 | 0.20 | -0.44 | -0.40 | -0.45 | 0.75* | -0.35 | -0.44 |
| γ-Tocopherol |  | 1 | 0.34 | 0.47 | 0.02 | -0.05 | -0.22 | 0.04 | 0.51 | 0.69 | 0.57 | -0.07 | 0.52 | 0.01 | 0.10 | 0.55 |
| Total tocopherol |  |  | 1 | 0.58 | -0.07 | 0.38 | -0.72* | 0.73* | 0.79* | 0.28 | 0.87** | 0.62 | 0.73* | -0.68 | 0.40 | 0.86** |
| PA.C16.0 |  |  |  | 1 | 0.65 | -0.38 | -0.81* | 0.24 | 0.36 | -0.13 | 0.59 | 0.27 | 0.56 | -0.65 | -0.06 | 0.57 |
| PA.C16.1 |  |  |  |  | 1 | -0.90** | -0.51 | -0.16 | -0.19 | -0.54 | -0.11 | 0.04 | -0.14 | -0.19 | -0.43 | -0.13 |
| SA.C18.0 |  |  |  |  |  | 1 | 0.17 | 0.44 | 0.36 | 0.44 | 0.34 | 0.15 | 0.29 | -0.15 | 0.55 | 0.35 |
| OA.C18.1 |  |  |  |  |  |  | 1 | -0.72* | -0.66 | 0.12 | -0.72* | -0.68 | -0.58 | 0.83* | -0.28 | -0.71* |
| LA.C18.2 |  |  |  |  |  |  |  | 1 | 0.85** | 0.17 | 0.76* | 0.84** | 0.58 | -0.72* | 0.71* | 0.77* |
| LA..C18.3 |  |  |  |  |  |  |  |  | 1 | 0.58 | 0.91** | 0.67 | 0.78* | -0.55 | 0.57 | 0.91** |
| GA..C20.1 |  |  |  |  |  |  |  |  |  | 1 | 0.43 | -0.13 | 0.42 | 0.22 | 0.13 | 0.42 |
| β-sitosterol |  |  |  |  |  |  |  |  |  |  | 1 | 0.63 | 0.91** | -0.72* | 0.57 | 1.00*** |
| Δ5-avenasterol |  |  |  |  |  |  |  |  |  |  |  | 1 | 0.44 | -0.65 | 0.72* | 0.65 |
| Campesterol |  |  |  |  |  |  |  |  |  |  |  |  | 1 | -0.72* | 0.34 | 0.92** |
| Cholesterol |  |  |  |  |  |  |  |  |  |  |  |  |  | 1 | -0.40 | -0.73* |
| Stigmasterol |  |  |  |  |  |  |  |  |  |  |  |  |  |  | 1 | 0.58 |

Table 2. Correlation coefficients between fatty acids, sterols and tocopherols

*^∗^P <* 0.05, *^∗∗^P <* 0.01 , *^∗∗∗^P <* 0.001 .
